# Supplementary material for: Family-Level Impact of Germline Genetic Testing in Childhood Cancer: A Multi Family Member Interview Analysis
Source: Cancers (Basel). 2025 Feb 4;17(3):517. doi: 10.3390/cancers17030517 (PMC11816119; doi:10.3390/cancers17030517)
Supplement: Supplementary file 1 [file cancers-17-00517-s001.zip › cancers-3371478-supplementary.pdf]

Supplementary Materials

Interview PARENT

"Genetic screening in childhood cancer: A study on families' experiences, resources and care needs"

Table S1. The interview guide below is a prototype for interviewing parents of a child with a blood disease or tumor where germline genetic testing has been initiated. The order of the questions depends on the input from the parent(s). The questions are asked in a very empathetic and sensitive manner, delving deeper into what the parent shares.

Process/Technique	Questions	Themes
**Opening questions**	**Introduction + ethical information**<br><br>*"Good day, Madam/Sir. My name is [your name], and I am one of the researchers involved in the project 'Genetic Testing in Childhood Cancer.' First of all, I want to thank you for agreeing to participate in this study. Without your help, this kind of research would not be possible. Your experiences and knowledge are invaluable to us.*<br>*With this study, we aim to understand the impact of germline genetic testing in childhood cancer on family members. Specifically, we want to investigate how parents and children experience genetic counseling and how it affects family life. We hope the results of this study will contribute to better support for families in similar situations.*<br>*As discussed on the phone, your participation involves both parents taking part in an interview. This conversation will be recorded with a voice recorder. Rest assured, this information will be handled with the utmost care and confidentiality. Only those directly involved in the study will have access to the recording. Later, a team member will transcribe the conversation, and all personal data will be pseudonymized. If your information is used for scientific articles, it will always be anonymized and unidentifiable.*<br>*During this interview, we will ask you several questions. It is important that you feel free to share only what you are comfortable sharing and what you feel is important to discuss. We encourage you to respect your boundaries and not feel pressured to talk about anything that makes you uncomfortable. You can pause or skip questions at any time.*	

	I will guide this conversation by asking some questions. Beyond that, I will mainly listen, as I want to understand your perspective and experiences as a parent as thoroughly as possible. After the interview, we will take some time to evaluate your experience of the conversation and your thoughts about the questions."<br><br>***[Go through informed consent + obtain signatures.]***<br>***[Remind them again of the options: pausing is possible, skipping questions is allowed.]***<br>***[Emphasize there are no right or wrong answers.]***	
START RECORDING	"I will now start the recorder so I can recall everything we discuss after the conversation."	
	**Short Questionnaire (Socio-Demographic Information)**<br><br>*"Before we start the interview, I would like to ask a few general questions about your background, such as age and education. Is that okay? All questions are listed on this short questionnaire, so feel free to fill it out."*	
**Introduction questions**	- What do you remember about the genetic testing?<br>**PROBES:**<br>Had you thought about heredity before your child's diagnosis?<br>What do you think is the right moment to offer genetic testing?<br>Were you, as parents, in agreement about starting genetic testing? How did the decision-making process go?	PERCEPTION<br>TIMING<br>DECISION-MAKING PROCESS
**Transition questions**	- How was it for you to initiate genetic testing?<br>**PROBES:**<br>How did you experience those months?<br>What emotions characterized this period?<br>How did you cope with the genetic testing and its results?	INDIVIDUAL MPACT<br><br>COPING
**Key questions**	Let's talk about the influence of genetic testing on your family. Could you start by describing what "family" means to you?	FAMILY IMPACT

	- To what extent has genetic testing affected your family (family members and family functioning)?<br><br>**PROBES:**<br>How did your family experience this period?<br>What changed in your family because of the genetic results?<br>How was genetic testing discussed within your family during this period?<br>Was there an impact on your relationship with your partner? To what extent?<br>Was there an impact on your relationship with the other parent? To what extent?<br>Was there an impact on your relationship with your sick child (diagnosed child)? To what extent?<br>Was there an impact on your relationship with your other children? To what extent?<br>Was there an impact on your relationship with others in your environment? To what extent?	COMMUNICATION<br><br>IMPACT ON PARTNER RELATIONSHIP<br>IMPACT PARENTHOOD<br><br><br>IMPACT CONTEXT
**Summary questions**	- What do you think is the most important takeaway from this conversation?<br>- Summarize: Is this correct? Did I miss anything?<br>- Are there any questions you would like to ask me? Or anything you would like to know?	
**Closing**	- Thank the parent(s) for participating.	
